# Going against the flow: bumblebees prefer to fly upwind and display more variable kinematics when flying downwind

**DOI:** 10.1242/jeb.245374

**Published:** 2023-04-18

**Authors:** Stacey A. Combes, Nick Gravish, Susan F. Gagliardi

**Affiliations:** ^1^ Department of Neurobiology, Physiology and Behavior, University of California, Davis, Davis, CA 95616, USA; ^2^ Department of Mechanical and Aerospace Engineering, University of California, San Diego, San Diego, CA 92161, USA

**Keywords:** Bee, Flight, Insect, Wind

## Abstract

Foraging insects fly over long distances through complex aerial environments, and many can maintain constant ground speeds in wind, allowing them to gauge flight distance. Although insects encounter winds from all directions in the wild, most lab-based studies have employed still air or headwinds (i.e. upwind flight); additionally, insects are typically compelled to fly in a single, fixed environment, so we know little about their preferences for different flight conditions. We used automated video collection and analysis methods and a two-choice flight tunnel paradigm to examine thousands of foraging flights performed by hundreds of bumblebees flying upwind and downwind. In contrast to the preference for flying with a tailwind (i.e. downwind) displayed by migrating insects, we found that bees prefer to fly upwind. Bees maintained constant ground speeds when flying upwind or downwind in flow velocities from 0 to 2 m s^−1^ by adjusting their body angle, pitching down to raise their air speed above flow velocity when flying upwind, and pitching up to slow down to negative air speeds (flying backwards relative to the flow) when flying downwind. Bees flying downwind displayed higher variability in body angle, air speed and ground speed. Taken together, bees' preference for upwind flight and their increased kinematic variability when flying downwind suggest that tailwinds may impose a significant, underexplored flight challenge to bees. Our study demonstrates the types of questions that can be addressed with newer approaches to biomechanics research; by allowing bees to choose the conditions they prefer to traverse and automating filming and analysis to examine massive amounts of data, we were able to identify significant patterns emerging from variable locomotory behaviors, and gain valuable insight into the biomechanics of flight in natural environments.

## Teaser

**Summary:** Data on thousands of flights from foraging bees show that bees prefer to fly upwind, and that body angle and speed become more variable when bees fly downwind.

## INTRODUCTION

Flying insects face numerous challenges in natural environments, including physical clutter and variable wind, and most insects rely heavily on visual feedback to stabilize themselves and navigate through complex landscapes (Taylor and Krapp, 2007). Our understanding of how insects accomplish these tasks is based primarily on laboratory studies in which insects are compelled to fly in a challenging scenario imposed by the researcher, such as maneuvering through obstacles (Crall et al., 2015; Lecoeur et al., 2019; Ravi et al., 2020), flying upwind through unsteady air flow (Crall et al., 2017; Ortega-Jiménez and Combes, 2018; Ortega-Jiménez et al., 2013; Ravi et al., 2013), or contending with clutter and wind simultaneously (Burnett et al., 2020). However, in outdoor settings, insects typically have some freedom to choose among alternative flight conditions; for example, by flying higher or lower to the ground, flying through or above obstacles, or altering their flight path to spend more time flying upwind (i.e. into a headwind), downwind (with a tailwind), or in crosswinds (along a path perpendicular to wind flow).

In addition to navigating these physical challenges, central-place foragers that fly over long distances in search of food require some mechanism of regulating their flight speed regardless of external wind and gauging the distance they have traveled, in order to return to their nest. Antennal sensing of air speed contributes to the regulation of flight speed in insects, particularly in the absence of strong visual cues (Khurana and Sane, 2016). But antennal sensing alone can only provide a measure of air speed (flight speed with respect to the surrounding air), and so provides inaccurate distance information if wind is present. Thus, many flying insects, including central-place foragers, rely strongly on visual mechanisms to control their ground speed (flight speed with respect to the ground) and measure the distance they have traveled.

Translational optic flow, or the angular velocity at which surrounding objects or surfaces move past an animal's eyes as it moves through the environment, can be used by flying insects in a variety of ways. When flying through corridors or obstacles, bees balance the translational optic flow on their left and right eyes to maintain position in the center of the corridor or gap (Kirchner and Srinivasan, 1989), and they use optic flow to estimate their distance from lateral walls or obstacles (Srinivasan et al., 1991). A variety of insects use optic flow to regulate air speed (reviewed in Baird et al., 2021), and fruit flies and bees also use optic flow to maintain constant ground speed when flying in the presence of wind (Baird et al., 2021; Barron and Srinivasan, 2006; David, 1982). Laboratory experiments have shown that honeybees (*Apis mellifera*) can maintain fixed ground speeds and optic flow in a variety of external flow conditions, including when flying upwind with headwinds greater than 3.5 m s^−1^ (Barron and Srinivasan, 2006) and when flying downwind with tailwinds up to 2 m s^−1^ (Baird et al., 2021). When flying upwind, bees increase their air speed beyond the velocity of the oncoming flow to maintain a preferred ground speed. Monitoring and controlling their ground speed allows bees to estimate the total distance they have flown, based on optic flow cues (Esch and Burns, 1995; Riley et al., 2003; Srinivasan et al., 1996).

Although bees are equally likely to encounter headwinds, tailwinds or crosswinds in natural environments, most laboratory-based flight studies (whether focused on sensory cues or flight kinematics) have focused on performance in still air or headwinds, as these conditions can most easily be simulated in the lab (e.g. by motivating insects to fly upwind in a wind tunnel). A few recent studies have explored honeybee flight in tailwinds (downwind) as well as headwinds (upwind), but the primary focus of these experiments was the role of visual cues (Baird et al., 2021) or the combined challenge of wind and physical obstacles (Burnett et al., 2020, 2022), rather than the effects of wind direction on the flight performance of bees. In addition, because insects are typically compelled to fly in a single environmental condition prescribed by the researcher, we do not know whether flying with wind coming from a particular direction is preferable to bees, whereas wind from other directions makes flight more challenging.

Data from studies on long-range migration or dispersal of insects provides some indirect information about insects' preferences for flight direction relative to wind. Radar studies reveal that many migrating insects rise far above the ‘flight boundary layer’ (FBL, i.e. the height at which wind speeds are approximately equal to the insect's own powered flight speed; Taylor, 1974), sometimes flying as high as 2–3 km above the surface. This presumably allows the insects to take advantage of strong winds that push them at speeds well beyond their maximum powered flight limits (reviewed in Chapman et al., 2011). Some of these migrating insects also display sophisticated height-selection strategies that allow them to adjust their altitude to fly with maximum tailwinds oriented in their intended direction of travel (Chapman et al., 2011). These studies on long-range windborne insect migrations show that migrating insects nearly always choose to fly downwind (i.e. with a tailwind).

However, a recent study on dispersal in *Drosophila melanogaster* suggests that flies do not simply fly downwind when released in a natural environment (Leitch et al., 2021). Instead, they choose a random direction of travel, then maintain a fixed heading (i.e. body orientation relative to celestial cues) while regulating their ground speed along their body axis, allowing them to be pushed sideways when external winds are not aligned with their flight heading. In this way, flies can disperse over large distances while maintaining the possibility of intercepting an odor plume that would lead them to an upwind food source (Leitch et al., 2021).

In a recent lab-based study on honeybee flight in headwinds and tailwinds, the authors reported that the wind speeds used in the study were limited to 2 m s^−1^ because this was the maximum speed at which bees would fly in a tailwind; in faster tailwinds, they would either land on the floor or exit the flight tunnel (Baird et al., 2021). This finding, along with the study on dispersal in fruit flies, suggests that insects' preference for flight direction relative to wind when they are flying within the FBL (i.e. within ∼0.5–15 m above the ground, where wind speed does not surpass powered flight capability) – a zone in which most insects spend the majority of their lives foraging and interacting with conspecifics – may differ from the preferences displayed by insects that engage in long-distance windborne migration above the FBL.

Here, we employed recent advances in automating video collection and analysis to examine thousands of foraging flights performed by hundreds of bumblebees flying in laboratory enclosures with both headwinds and tailwinds. We developed two novel experimental approaches to examine bumblebee flight in headwinds (upwind) versus tailwinds (downwind), in an effort to answer three questions about these commonly experienced flight conditions: (1) do bumblebees display a preference for flying upwind or downwind?; (2) do bumblebees maintain constant ground speed when flying downwind, as they do when flying upwind?; and (3) do bees display similar flight kinematics when flying upwind and downwind, or do these conditions impose different aerodynamic challenges?

## MATERIALS AND METHODS

### Experiment 1

#### Two-choice flight arena

**Fig. 1.**
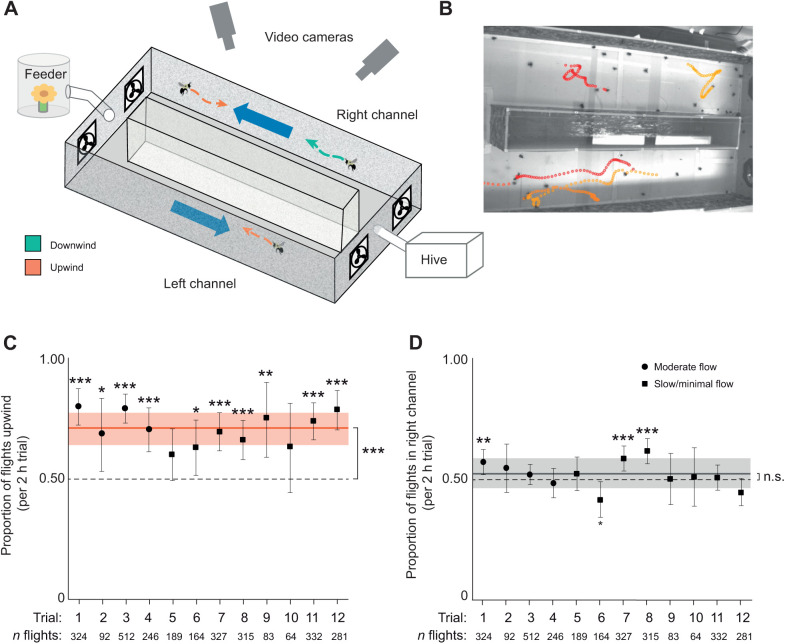
**Bees choose to fly upwind more often than downwind.** (A) The two-choice flight arena used in experiment 1, in which bees could choose to fly from their hive to a feeder (and back to their hive) via one of two channels, with wind flowing in opposite directions. Flights were analyzed over 1.2 s video clips (filmed at 50 frames s^−1^) captured every minute over a 2 h period each day. (B) Image from one camera view of the flight arena, with several 1.2 s flight paths highlighted that were retained for analysis after removing walking bees. (C) Proportion of flights that occurred in the upwind (as opposed to downwind) direction. Over 12 days of testing, 2929 flights were recorded. The mean (±s.d.) proportion of bees flying upwind was 0.644±0.046, which was significantly greater than 0.5 (Wilcoxon test, *P*=0.00024). The number of flights recorded during each 2 h trial (*n*) is shown below the *x*-axis, and the different wind conditions are shown by different symbols (moderate, 1.25 m s^−1^; slow, 1.07 m s^−1^; minimal, 0.25 m s^−1^). (D) Proportion of flights that occurred in the right channel (as opposed to the left channel). The mean proportion of bees flying in the right channel was 0.525±0.060, which was not significantly greater than 0.5 (Wilcoxon test, *P*=0.076). In C and D, asterisks show results of binomial tests to determine whether each day's proportion of flights was significantly different from 0.5 (*P<0.05, **P<0.01, ***P<0.001; n.s., not significant). The solid, horizontal line shows the mean proportion over 12 days of testing, and shading shows ±1 s.d.

In the first part of our study (experiment 1), we constructed a two-choice flight arena, in which a hive of yellow-faced bumblebees (*Bombus vosnesenskii* Radoszkowski 1862) could fly from their hive at one end to a feeder at the opposite end, which they could access via two different flight channels (Fig. 1A). The feeder contained the colony's only source of nectar (which was unscented, 50% sugar water, *ad libitum*); pollen was provided within the hive. Each flight channel was approximately 20×20 cm in cross-section and 1 m long, and the walls were covered in a speckled pattern to provide visual cues. Bees were allowed to acclimate to foraging in the arena for 1 week before experiments began, so that they would be familiar with the location of the feeder, the hive and the two channels.

We created air flow along each channel by embedding computer fans at both ends, with both fans blowing in the same direction (i.e. with one fan pushing air in from one end while the other fan simultaneously pulled air out from the other end). Within each channel, we could reverse the direction of flow by physically removing and re-installing the fans on each end so that they moved air in the opposite direction. In all trials, air flowed in opposite directions in the two channels (i.e. one channel had air flowing from hive to feeder and the other had air flowing from feeder to hive, with the direction in each channel varied on different days). In some trials, we turned on the fans in both channels, to create flows of moderate velocity (1.25 m s^−1^) in opposite directions. In other trials, we only turned on the fans in one channel, which led to slightly slower flow (1.07 m s^−1^) in that channel, along with minimal flow (0.25 m s^−1^) in the opposite direction in the other channel (due to some air circulation between channels through the open, end sections where both channels ended; Fig. 1A).

We systematically varied the direction of flow in the two channels to determine whether bumblebees display a consistent preference for flying upwind or downwind, while controlling for any preference the bees may have for flying in one channel versus the other (designated the ‘left’ and ‘right’ channels), or for any potential differences in flow characteristics or turbulence level between the channels (which we believe were minimal, because of the lack of obstructions within channels and the low flow velocity).

For each foraging trip an individual made, they were presented with two separate choices, deciding which tunnel to fly in for the trip from the hive to the feeder, and then deciding which tunnel to fly in for the return trip from the feeder to the hive. Experiments were performed over 12 days, and a single flow condition was tested on each day. Bees were allowed to acclimate to the new flow condition for 1 h before data collection began. We tested 6 different experimental conditions in randomized order, with 2 days/recording sessions per condition: (1) moderate flow (1.25 m s^−1^ in both channels), with flow in the left channel towards the feeder (and flow in the right channel towards the hive), (2) moderate flow, with flow in the left channel towards the hive, (3) slow/minimal flow (1.07 m s^−1^ and 0.25 m s^−1^) with slow flow in the left channel towards the feeder (and minimal flow in the right channel towards the hive), (4) slow/minimal flow with slow flow in the left channel towards the hive, (5) slow/minimal flow with slow flow in the right channel towards the feeder, and (6) slow/minimal flow with slow flow in the right channel towards the hive.

After each day's hour-long acclimation period, we collected video data over a period of 2 h (from noon to 14:00 h), recording a subsample of 1.2 s of video per minute (resulting in 120 flight clips per recording session). The entire length of both channels was filmed using two synchronized video cameras (Photonfocus MV1-D1312-160-CL) along the length of the arena, recording at 50 frames s^−1^. Cameras were calibrated each day using a checkerboard calibration routine in Matlab, and were automated to start, stop and save 1.2 s video clips every minute throughout the recording session.

#### Video analysis and statistical testing

Video data were analyzed in Matlab using motion-based multiple object tracking. This involved background subtraction to detect moving bees and a Kalman filter to assign moving points (bees) to tracks. Note that individual bees could not be uniquely identified because of the wide view of the filming area and subsequent low resolution of each individual. Given the large number of flights analyzed (which was substantially higher than the number of workers normally present in a hive) and the fact that some individuals within bumblebee hives are known to perform more foraging flights than others (Crall et al., 2018), our dataset is assumed to contain repeated measures of multiple flights by individual bees, which increases the chance of Type 1 statistical errors (see Discussion). Short tracks (less than 6 frames long) and erroneous points (points that became stationary) were removed, and we created 3D flight paths by matching tracks from different cameras and minimizing residual error (Fig. 1B). The 3D flight paths allowed us to exclude bees whose entire track was less than 1.5 cm above the floor of a channel (and thus were assumed to be walking) from further analysis.

We pooled all flights within each 2 h filming session, and classified each flight as upwind or downwind, and as left channel or right channel, depending on the location of the bee, the direction of its motion, and the direction of air flow during that trial. We then summed the total number of flights that were upwind and divided by the total number of flights to calculate the proportion of upwind flights (note that this total includes flights in both the left and right channels, as flow was upwind in each channel for one of the directions of travel, from hive to feeder or feeder to hive). We separately summed the total number of flights in the right channel (regardless of flow direction) and divided by the total number of flights to find the proportion of flights in the right channel.

Using the proportions calculated for each of the 12 days of data collection, we tested whether the proportion of upwind flights (and separately whether the proportion of flights in the right channel) was significantly greater than 0.5, using a one-sample Wilcoxon test in R (one-sided test to determine whether the proportion is greater than 0.5, *n*=12 days/proportions). Finally, because the total number of bees foraging each day can vary substantially (this is typical, and is seen even in the absence of experimental treatments), we tested each day's proportion of upwind (and right channel) flights to determine whether it was significantly different from 0.5 using a two-sided binomial test in R.

### Experiments 2 and 3

#### Wind tunnel foraging experiments

**Fig. 2.**
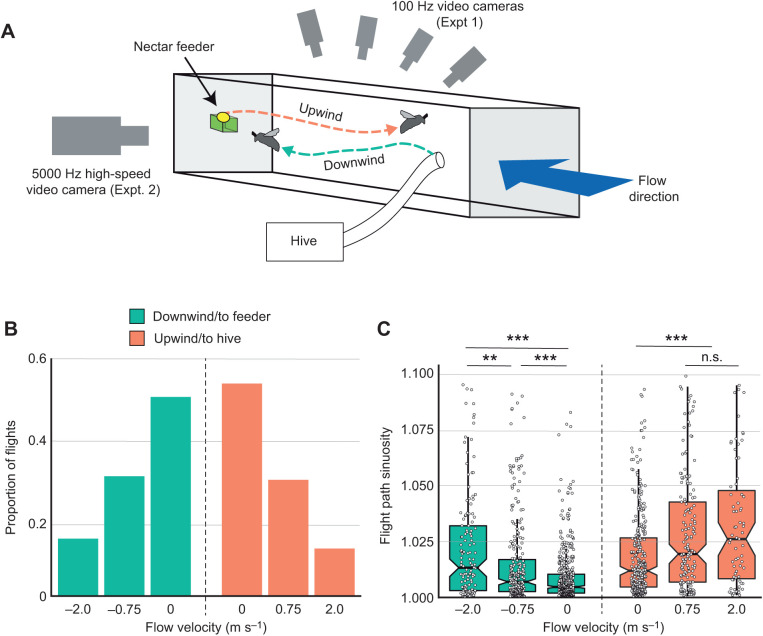
**Bees fly less frequently and along more sinuous flight paths in higher flow velocities.** (A) In wind tunnel experiments, bees were allowed to fly freely from a hive entrance at the upstream end of a wind tunnel working section to a feeder at the downstream end, flying downwind from the hive to the feeder and upwind from the feeder to the hive. Flow velocities were alternated for hour-long periods between 0, 0.75 and 2.0 m s^−1^, and bees were filmed with either four 100 Hz cameras over the working section (experiment 2) or one 5000 Hz camera capturing a lateral view (experiment 3). (B) The proportion of total flights recorded in experiment 2 was highest during periods with no flow (0 m s^−1^) and lowest during periods with 2.0 m s^−1^ flow. Proportions were calculated separately for downwind and upwind flights. A total of 1662 flights were captured over 6 days, with three hour-long periods of filming each day. (C) Flight path sinuosity (total distance traveled divided by linear distance from the start to the end point) in experiment 2 increased with flow speed, for bees traveling in both directions. Notched box plots show the median, 25th and 75th percentiles, and circles show individual data points. Upwind and downwind flights were analyzed separately (see Materials and Methods); asterisks indicate significant differences (one-way ANOVA with Tukey's HSD, **P<0.01, ***P<0.0001; n.s., not significant).

In the second part of our study (experiments 2 and 3), we allowed a hive of common eastern bumblebees (*Bombus impatiens*; Cresson 1863) to forage freely over a period of several weeks at a nectar feeder placed in the working section of a wind tunnel, traveling round-trip to the feeder from the exit/entry of their hive at the other end of the working section. As in experiment 1, individual bees could not be uniquely identified, and our dataset is assumed to contain repeated measures of multiple flights by individual bees, which increases the chance of Type 1 statistical errors (see Discussion). Bees encountered tailwinds when flying from the hive to the feeder, and headwinds when returning from the feeder to the hive (Fig. 2A). The working section of the wind tunnel was 45×45 cm in cross-section and 1.4 m long. Flow within the tunnel was unimpeded by the feeder (as this was at the downstream end of the working section), and turbulence intensity was low (<1.2%; Ravi et al., 2013). Black vertical bars 1 cm in width and spaced 2 cm apart were printed on clear film and attached to the side walls of the working section to provide visual cues. Bees were allowed to freely enter and exit the working section via a tube connecting the wind tunnel to their hive. The feeder on the downwind side of the working section provided *ad libitum* artificial nectar (50% sugar water) and was the only source of nectar for the hive; pollen was provided within the hive.

We performed experiments 2 and 3 in the wind tunnel on two separative hives of bumblebees. In experiment 2, we filmed bees with four overhead video cameras (Photonfocus MV1-D1312-160-CL), which imaged overlapping regions covering the full length of the working section, to obtain recordings of bees' overall flight velocities and trajectories while traveling upwind or downwind. Videos were motion triggered throughout the filming period and recorded at 100 Hz. Flow velocity was varied over three levels: 0, 0.75 and 2 m s^−1^. We allowed bees to acclimate to the wind tunnel for 3 days prior to performing wind experiments. The three flow velocity treatments were presented each day between the hours of 13:00 h and 16:00 h, and each treatment lasted for 1 h. We performed flight trajectory experiments over 6 days and modified the order of treatments to account for all possible combinations.

In experiment 3, we used a high-speed video camera (Phantom v410, Vision Research) to capture high-resolution videos at 5000 Hz, to analyze details of bees' body and wing kinematics during upwind and downwind flights. The high-speed camera was placed on the side of the wind tunnel to capture a lateral view of bees flying upwind or downwind, and a calibration object was used to convert video data from pixels to centimeters. The camera filmed an area of 10×10 cm, and was automatically triggered by bees flying through a laser aimed at a photoresistor. In this experiment, we varied flow velocity over the same three levels (0, 0.75 and 2.0 m s^−1^) throughout the day over the course of 2 weeks, performing additional trials at some velocities until enough video clips in each condition were captured.

#### Video analysis and statistical testing

Video data from both wind tunnel experiments were tracked using custom code in Python that incorporated the OpenCV package (https://github.com/nickgravish/Tracker). The image processing procedure consisted of: (1) computing the background from the median pixel values over time, (2) background removal and thresholding to isolate foreground objects (i.e. bees), (3) contour identification and ellipse fitting of foreground objects. After these processing steps, we had a set of bee contours (ellipses) for every video frame. In the next step, we performed contour association to link bee observations across frames. This step is unnecessary when there is only one bee in the video; however, in cases where multiple bees are present (which did occur), this is a necessary step to properly link tracks across video frames. To perform data association, we used a modified Kalman filter that linked objects across frames by minimizing the positional error between frames. This association step resulted in a list of flight track information for each frame, including body position and orientation (from the fitted ellipse), body size (from the number of thresholded pixels and a pixel to centimeter calibration), and velocity (estimated for each frame as the output of the Kalman filter). The final video processing step was to refine body orientation by removing fast-moving objects (the wings) and retaining slow-moving objects (the body).

From this flight track information, we calculated several kinematic variables. For experiment 2 (flight paths viewed from above), we restricted our analysis to trajectories within the central 30 cm of the tunnel's length, during which all bees were in motion (i.e. not taking off or landing). We calculated the sinuosity of each flight trajectory as the total distance along the 2D flight path divided by the linear distance between the start and end points of the trajectory. We noted that in a small number of flights, bees reversed direction, flew in a loop, or performed other maneuvers that interrupted their progress from one end of the tunnel to the other, resulting in high path sinuosity. Bees flying along more sinuous paths would experience varied, fluctuating optical flow, which could affect our comparison of optic flow regulation in upwind versus downwind flights; thus, we removed flights with high sinuosity (defined as sinuosity >1.1) and restricted our analyses to relatively direct flights with path sinuosity of 1.1 or less. We also excluded trajectories in which mean ground speed (see below) was less than 0.02 m s^−1^, as these likely represented bees walking on the bottom of the working section rather than flying (speeds along the tunnel were bimodal, with the low-speed peak occurring below 0.02 m s^−1^).

From the remaining trajectories, we calculated the mean and standard deviation of ground speed (the bee's speed relative to the ground, regardless of flow velocity), based on the instantaneous speed of the bee along the tunnel's long axis (i.e. speed along the *x*-axis, defined as the dimension aligned with the walls of the tunnel). We also calculated the mean and standard deviation of air speed (the bee's speed relative to the surrounding air), by adding the flow velocity to the bee's ground speed (when bees were flying upwind) or subtracting the flow velocity from the bee's ground speed (when bees were flying downwind).

To determine whether flights from the hive to the feeder (downwind when flow was present) and from the feeder to the hive (upwind with flow) could be analyzed together, we used a two-sample Wilcoxon test to compare bees' mean ground speed, standard deviation of ground speed, and sinuosity of flights in the two directions with 0 m s^−1^ air flow. Based on the outcome of these tests (see Results), we performed further analyses on flights in the two directions separately. To determine how flow velocity in the wind tunnel affected the measured kinematic variables, we performed one-way ANOVA on each variable (mean and standard deviation of bees' ground speed, mean and standard deviation of bees' air speed, and path sinuosity) with flow velocity (0, 0.75 or 2 m s^−1^) as a factor, analyzing flights from the hive to the feeder (the ‘downwind’ direction) and flights from the feeder to the hive (the ‘upwind’ direction) separately. *Post hoc* testing for significant variables was performed with Tukey's HSD test. Because some of the data did not meet assumptions of normality and homogeneity of variance, we also performed an equivalent non-parametric test on each set of data (a Kruskal–Wallis chi-squared test) to verify our results.

For experiment 3 (lateral high-speed videos), we used the orientation of ellipses fitted to the bees' bodies to calculate pitch angle, as the angle between the body axis and the horizontal. For each trajectory, we found the mean body pitch angle as well as the standard deviation of body angle. Finally, we calculated the average flapping frequency for each flight by measuring the frequency component of the instantaneous velocity along the tunnel axis (the *x*-axis). The velocity along this axis is calculated from the lateral bee silhouette, which has a slow component associated with center of mass movement and acceleration, and a fast component associated with the rapid forward and backward shift of the silhouette due to the wing motion. We performed a fast Fourier transform (FFT) on the *x*-velocity time series and determined the frequency of the maximum power signal of the FFT to estimate flapping frequency.

As in experiment 2, we tested the data to determine whether flights from the hive to the feeder and from the feeder to the hive differed, by performing a two-sample Wilcoxon test to compare mean body pitch angle, standard deviation of body angle, and mean flapping frequency in the two directions with 0 m s^−1^ air flow. Based on the outcome of these tests (see Results), we performed further analyses on flights in the two directions separately. To determine how flow velocity in the wind tunnel affected the measured kinematic variables, we performed one-way ANOVA on each variable (mean and standard deviation of body angle, mean flapping frequency) with flow velocity (0, 0.75 or 2 m s^−1^) as a factor, analyzing flights from the hive to the feeder and from the feeder to the hive separately. Because some of the data did not meet assumptions of normality and homogeneity of variance, we also performed an equivalent non-parametric test on each set of data (a Kruskal–Wallis chi-squared test) to verify our results.

## RESULTS

### Two-choice flight arena

Our automated methods of video collection and analysis in experiment 1 allowed us to examine 2929 voluntary foraging flights (both outbound and return flights to the hive) in the two-choice flight arena over 12 days of filming, with foraging sub-sampled over a 2 h period each day. This included 804 flights with moderate flow velocity (1.25 m s^−1^) in both channels, and 1117 flights with low flow velocity (1.07 m s^−1^) in one direction and minimal flow velocity (0.25 m s^−1^) in the other direction. The total number of flights recorded over the testing period varied between days, from a minimum of 64 to a maximum of 512 (mean±s.d. 244±132 flights day^−1^; Table S1). Based on the proportions calculated for each of the 12 days of data collection, we found that the mean proportion of bees flying upwind was 0.644±0.046, and the overall proportion of bees flying upwind was significantly greater than 0.5 (one-sample Wilcoxon test, *V*=78, *P*=0.00024; Fig. 1C). In contrast, the mean proportion of bees flying in the right channel was 0.525±0.060, which was not significantly greater than 0.5 (one-sample Wilcoxon test, *V*=58, *P*=0.076; Fig. 1D). The binomial tests to determine whether each day's proportion of flights was significantly different from 0.5 showed that the proportion of bees flying upwind was significantly greater than 0.5 on 10 of the 12 days (Fig. 1C; Table S1). In contrast, the proportion of bees flying in the right channel was not significantly different from 0.5 on 8 of the 12 days, was significantly higher than 0.5 on 3 days, and was significantly lower than 0.5 on 1 day (Fig. 1D; Table S1).

### Wind tunnel foraging experiments

Experiment 2, in which we captured overhead views of flight trajectories along the wind tunnel, resulted in 1662 digitized trajectories over 6 days (with motion-triggered videos collected over a period of 3 h per day). After excluding high-sinuosity flights and low-speed walking tracks, we had a total of 1449 flights for analysis. These included 470 flights towards the feeder with 0 m s^−1^ flow, 283 flights towards the feeder with a 0.75 m s^−1^ tailwind, and 136 flights towards the feeder with a 2 m s^−1^ tailwind, as well as 316 flights towards the hive with 0 m s^−1^ flow, 173 flights towards the hive with a 0.75 m s^−1^ headwind, and 71 flights towards the feeder with a 2 m s^−1^ headwind. Despite filming bees for the same total amount of time at each flow velocity, we found that the number of flights declined sharply as flow velocity increased; thus, more than 50% of the flights captured in each direction occurred with no flow (0 m s^−1^) and fewer than 20% of flights occurred in 2 m s^−1^ flow (Fig. 2B).

We found that bees' flight behavior differed significantly when flying down the wind tunnel towards the feeder and when flying up the tunnel to return to the hive, even in the absence of external flow. Flight trajectories with no flow (0 m s^−1^) differed significantly between the two directions in mean ground speed (two-sample Wilcoxon test, *P*=6.6×10^−7^) and path sinuosity (*P*<2.2×10^−16^), although the standard deviation of ground speed was not significantly different (*P*=0.76). We therefore analyzed flights in the two directions separately.

**Fig. 3.**
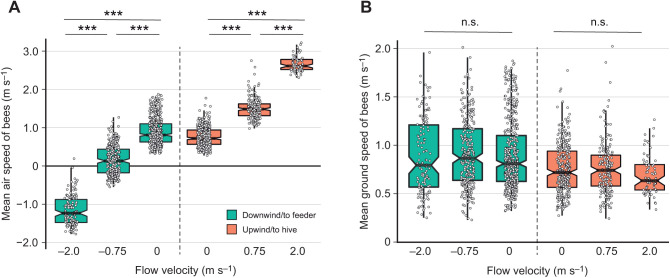
**Bees' air speed differs but ground speed is unaffected by flow velocity.** (A) Bees' air speed (flight speed relative to the surrounding flow) increased with stronger upwind flow velocities, and decreased with stronger downwind flow velocities, reaching negative values in 2.0 m s^−1^ tailwinds (i.e. bees flew backwards relative to the flow). (B) Bees' ground speed (flight speed relative to the ground) remained the same for upwind flights at all flow velocities, and for downwind flights at all flow velocities. Data for both panels are from experiment 2, conducted in a wind tunnel (*n*=1629 flights). Notched box plots show the median, and 25th and 75th percentiles, and circles show individual data points. Upwind and downwind flights were analyzed separately; asterisks indicate significant differences (one-way ANOVA with Tukey's HSD, ***P<0.0001; n.s., not significant).

When flying in both the downwind and upwind directions, bees' flight path sinuosity was affected by flow velocity (Table S2), with increased sinuosity in higher flow velocities (Fig. 2C). Bees' mean air speed also varied with flow velocity, in both the downwind and upwind directions (Table S2). Air speed increased significantly with flow velocity for bees flying upwind and decreased significantly with flow velocity for bees flying downwind, with bees in 0.75 m s^−1^ flow displaying airspeeds averaging around 0 m s^−1^ and bees in 2.0 m s^−1^ flow displaying negative air speeds (i.e. flying backwards relative to the air; Fig. 3A). Despite these large changes in bees' air speed, their mean ground speed was unaffected by flow velocity, for flights in either the upwind or downwind directions (Table S2; Fig. 3B).

In experiment 3, in which we captured lateral, high-speed videos of bees flying upwind or downwind, our automated triggering system allowed us to capture 457 high-resolution, 5000 Hz videos over the course of 2 weeks. These included 151 flights towards the feeder with 0 m s^−1^ flow, 98 flights towards the feeder with a 0.75 m s^−1^ tailwind, and 32 flights towards the feeder with a 2 m s^−1^ tailwind, as well as 98 flights towards the hive with 0 m s^−1^ flow, 61 flights towards the hive with a 0.75 m s^−1^ headwind, and 17 flights towards the flight with a 2 m s^−1^ headwind. One flight was excluded from analysis because it was an extreme outlier (standard deviation of body angle was ∼6 times higher than the mean).

As in experiment 2, we found that bees' flight kinematics differed significantly when flying down the wind tunnel towards the feeder and when flying up the tunnel to return to the hive, even in the absence of external flow. Flights with no flow (0 m s^−1^) differed significantly between the two directions in mean body angle (two-sample Wilcoxon test, *P*=5.917×10^−11^) and flapping frequency (*P*=5.027×10^−11^), although standard deviation of body angle was not significantly different (*P*=0.4499). We therefore analyzed flights in the two directions separately.

**Fig. 4.**
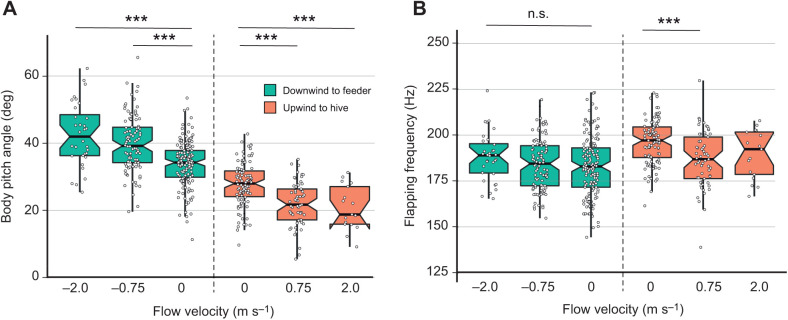
**Flow velocity strongly affects bees' body angle, but not flapping frequency.** (A) Bees displayed significantly lower body pitch angles during upwind flights at 0.75 and 2.0 m s^−1^, and higher pitch angles during downwind flights at 0.75 and 2.0 m s^−1^. (B) The flapping frequency of bees' wings was unaffected by flow velocity in the downwind direction, and differed only between 0 and 0.75 m s^−1^ in the upwind direction. Data for both panels are from experiment 3, conducted in a wind tunnel (*n*=457 flights). Notched box plots show the median, and 25th and 75th percentiles, and circles show individual data points. Upwind and downwind flights were analyzed separately; asterisks indicate significant differences (one-way ANOVA with Tukey's HSD, ***P<0.0001; n.s., not significant).

Body angle varied systematically with flow velocity, with bees displaying lower body angles when flying towards the hive in 0.75 and 2.0 m s^−1^ headwinds than when flying towards the hive in 0 m s^−1^ flow (Fig. 4A; Table S2). This pattern continued for flights towards the feeder, but with bees displaying higher body angles in 0.75 m s^−1^ and 2.0 m s^−1^ tailwinds than when flying towards the feeder in 0 m s^−1^ flow (Fig. 4A; Table S2). Flapping frequency, in contrast, varied little with flow velocity (Fig. 4B; Table S2). There was no difference in flapping frequency for flights towards the feeder (downwind direction); for flights towards the hive, frequency differed only between 0 m s^−1^ flights (196.2±12.1 Hz) and 0.75 m s^−1^ flights (186.9±15.1 Hz; Table S2).

**Fig. 5.**
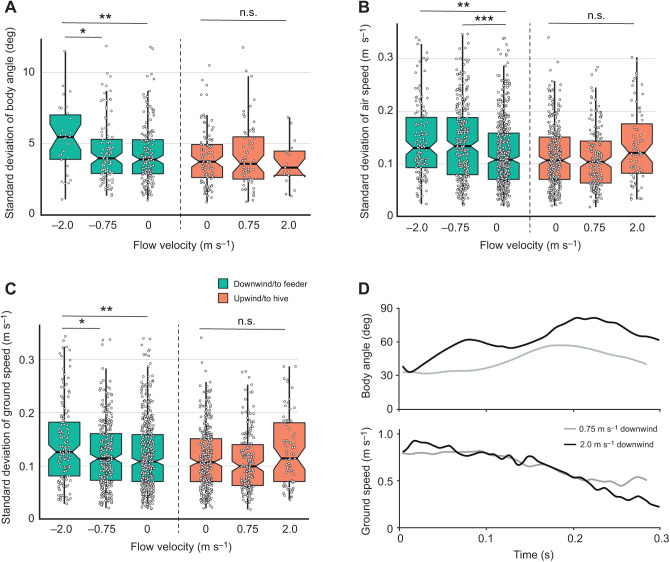
**Bees flying downwind display more variable body angles, air speeds and ground speeds as flow velocity increases.** (A) The standard deviation of body angle (i.e. variability in body angle within individual flights, averaged over all flights) was significantly higher for bees flying downwind in 2.0 m s^−1^ flow compared with 0.75 m s^−1^ flow or no flow. Standard deviation of body angle did not vary with flow velocity for flights in the upwind direction. (B) The standard deviation of bees' air speed was significantly higher when flying downwind in 0.75 or 2.0 m s^−1^ flow as compared with still air, but did not differ for flights in the upwind direction. (C) The standard deviation of bees' ground speed was significantly higher when flying downwind in 2.0 m s^−1^ flow compared with 0.75 m s^−1^ flow or no flow, but did not differ for flights in the upwind direction. (D) Sample data from one downwind flight with 0.75 m s^−1^ flow (gray) and one with 2.0 m s^−1^ flow (black), showing variation in body angle (top) and ground speed (bottom) throughout the flight. Data for A and D are from experiment 3 (*n*=457 flights), and those for B and C are from experiment 2 (*n*=1629 flights). Notched box plots show the median, and 25th and 75th percentiles, and circles show individual data points. Upwind and downwind flights were analyzed separately; asterisks indicate significant differences (one-way ANOVA with Tukey's HSD, *P<0.05, **P<0.01, ***P<0.0001; n.s., not significant).

The average standard deviation of bees' body angle (i.e. how much body angle varied within flights) was significantly higher in 2.0 m s^−1^ downwind flights than in 0.75 or 0 m s^−1^ flights in the downwind direction (Fig. 5A; Table S2), but there was no difference in the upwind direction. Similarly, the standard deviation of bees' air speed was higher in 2.0 and 0.75 m s^−1^ downwind flights than in 0 m s^−1^ flights in the downwind direction (Fig. 5B; Table S2), but there was no difference in the upwind direction. In addition, the standard deviation of bees' ground speed was higher in 2.0 m s^−1^ downwind flights than in 0.75 or 0 m s^−1^ flights in the downwind direction (Fig. 5C; Table S2), but there was no difference in the upwind direction.

## DISCUSSION

### Preference for flying upwind versus downwind

Our novel two-choice flight arena and the automated filming and analysis methods we employed allowed us to capture and analyze nearly 3000 flights (and thus 3000 choices between the two channels) over 12 days of filming. The results show that foraging bees do not display a preference for flying downwind, as has previously been shown in studies on migrating insects and birds. In birds, flying with a tailwind can lead to considerable energetic savings (Alerstam, 1979; Butler et al., 1997), and many species display a preference for flying with a tailwind during migration (Åkesson and Hedenström, 2000; Dänhardt and Lindström, 2001; Green, 2004). Similarly, radar studies of migrating insects that engage in long-range windborne migration show that these insects nearly always fly downwind, and appear to preferentially select flight altitudes that provide them with the fastest downwind flow speed oriented in their direction of travel (reviewed in Chapman et al., 2011). In behavioral contexts outside of migration, however, flight behavior may be driven by additional factors beyond energetics; for example, when dispersing in natural habitats, fruit flies adopt a set heading relative to celestial cues and maintain a fixed ground speed along their body axis, covering less total distance than if they flew downwind, but maintaining the possibility of intercepting odor plumes from upwind food sources. In addition, Ellington et al. (1990) found that the energetic cost of flight for bumblebees flying in headwinds from 0 to 4 m s^−1^ was not strongly affected by wind speed. Thus, the energetic cost of flight in headwinds is unlikely to be a factor affecting bees' preferences for wind direction, as is the case for other migrating animals.

Rather than being impartial about the orientation of wind relative to their flight path (i.e. choosing randomly between the two channels), we found that bumblebees display a consistent preference for flying upwind (Fig. 1C), even when flow velocities are very low (0.25–1.25 m s^−1^). We eliminated the possibility that our data were affected by a preference for one of the tunnels itself (i.e. for the left versus right tunnel) by alternating the direction of flow in the two tunnels and analyzing the proportion of flights that occurred in the left versus right tunnel (Fig. 1D). The mean proportion of flights occurring in the right tunnel (averaging proportions calculated each day over the 12 days of the study) was not significantly greater than 0.5, indicating that bees had no preference for one tunnel over the other. In contrast, approximately 65% of the 2929 flights occurred in headwinds, and the mean proportion of flights in headwinds was significantly greater than 0.5.

Identifying this consistent preference would likely not have been possible by performing flight trials or choice tests on individuals one by one, as individual flight behavior tends to be highly variable in bumblebees, both across individuals and over different trials. However, our bulk-data approach of sub-sampling the flight choices of an entire hive of bumblebees presented with a two-choice paradigm over several weeks allowed us to collect enough data to identify this preference, despite high behavioral variability.

It should be noted that our inability to uniquely identify individual bees (and thus to account statistically for repeated measures) and the large sample sizes we were able to collect using automated techniques increase the likelihood of Type 1 statistical errors (in which the null hypothesis is erroneously rejected), in both the two-choice flight arena study (experiment 1) and the wind tunnel studies (experiments 2 and 3). The challenge of automatically recognizing and re-identifying individuals over multiple days, and of analyzing large datasets in ways that reduce the likelihood of Type 1 statistical errors, is an area of ongoing research that deserves further attention (and will be discussed in more detail below). Some studies suggest that lowering the critical *P*-value below *P=*0.05 can help reduce the likelihood of Type 1 errors in analyses of large datasets; the *P*-value for the majority of our results was in fact far below *P*=0.05, and often many orders of magnitude below *P*=0.01 (see Tables S1 and S3). Although we cannot rule out the possibility of a Type 1 error, given the number of days over which we collected and analyzed data, and the very low *P*-values that we obtained, we are confident that we have identified a true preference for flying upwind in the current study.

One possible explanation for why bees prefer to fly upwind could be that flying upwind provides bees with a rich source of olfactory information about the environment they are flying towards, whereas olfactory cues that a bee receives when flying downwind are far less informative. Olfactory cues are likely to be more prevalent than visual cues when bees are searching for new patches of flowers (Sprayberry, 2018), and several lab-based studies have shown that bumblebees can navigate towards floral resources using odor alone (Sprayberry et al., 2013; Spaethe et al., 2007). Field studies on honeybees have shown that honeybee recruits require odor to localize food sources, and feeding stations located downwind of hives have the longest search times and the lowest recruit success rates (Friesen, 1973). To reduce the chances of olfactory information affecting our results, we used unscented nectar in the foraging arena and supplied pollen directly to the hive. In addition, the two-choice flight arena is relatively small (total area <1 m^2^, with flight tunnels ∼1 m in length), the nectar source and location of the hive entrance were never changed, and bees had ample time to become acquainted with the arena and these locations before the experiment began, which makes the use of olfactory cues in search behavior less important for bees in this context. Nonetheless, we cannot entirely eliminate the possibility that bees have an innate preference for flying upwind (into a headwind) because of the enhanced olfactory information that this behavior provides.

### Regulation of ground speed

Our results show that bumblebees are capable of maintaining fixed ground speeds (and thus optic flow) when flying in tailwinds as well as headwinds, over flow velocities ranging from 0 to 2.0 m s^−1^ (Fig. 3B), which agrees with recent findings for honeybees (Baird et al., 2021). Bees' ground speeds when flying in the upwind direction of the wind tunnel (from feeder to hive) were slightly lower (means from 0.69 to 0.77 m s^−1^) than when flying in the downwind direction (from hive to feeder, means from 0.89 to 0.91 m s^−1^; Table S2), but because this difference was present even with no external flow, we interpret this as being due to different behavioral motivations and/or loading states when bees were traveling in these directions.

When flying in a given direction within the wind tunnel, bees' ground speeds did not differ significantly with external flow velocity (Fig. 3B) and, as expected, bees displayed large changes in air speed as flow velocity and direction changed (Fig. 3A). These changes in air speed result from a combination of the imposed external flow and bees' adjustments of their flight kinematics to maintain a preferred ground speed. Because bees' preferred ground speeds in this setting (tunnel with a width of 45 cm) ranged from 0.7 to 0.9 m s^−1^ on average, they increased their air speed beyond that of the external flow when flying into a headwind, such that their average air speed varied from 0.76 m s^−1^ with no flow to 2.7 m s^−1^ in 2.0 m s^−1^ headwinds (Fig. 3A). In contrast, to maintain constant ground speed in the downwind direction, bees decreased their airspeed relative to the external flow, such that their air speed dropped to an average of only 0.15 m s^−1^ with 0.75 m s^−1^ tailwinds and to −1.17 m s^−1^ with 2.0 m s^−1^ tailwinds – meaning that bees were flying backwards with respect to the surrounding flow, in order to slow themselves down enough to maintain their preferred ground speed.

Bees appear to have accomplished this control over air speed primarily by adjusting the pitch angle of their bodies (Fig. 4A). Previous wind tunnel experiments with bumblebees revealed a high correlation between body pitch and headwind speed (Dudley and Ellington, 1990). These results suggest that speed regulation may be controlled by bees in a manner similar to helicopters, by pitching forward (nose down) to tilt the net force production vector in a more forward direction and increase air speed, and by pitching up to reduce the forward tilt of the force vector and reduce air speed. Our results provide further support for this method of flight speed control in bumblebees, showing that bees not only pitch down to increase their air speed in headwinds but also pitch up to decrease their air speed in the presence of tailwinds (from a mean of 33.8 deg with no flow to 42.4 deg with 2.0 m s^−1^ tailwinds; Fig. 4A), to the point where their net force production vector is directed backwards, opposite to the direction in which they are traveling.

We also found that bees' wingbeat frequency does not increase significantly as headwind or tailwind flow velocity rises (Fig. 4B). Previous studies on bumblebees have shown that the energetic cost of flight (measured by O_2_ consumption) does not vary for bees flying in headwinds ranging from 0 to 4.0 m s^−1^ (Ellington et al., 1990), and studies of loaded flight (with no external flow) suggest that flapping frequency is the primary determinant of the energetic cost of flight in bees (measured by CO_2_ output; Combes et al., 2020). Thus, our finding that flapping frequency does not change across headwind and tailwind flow velocities from 0 to 2 m s^−1^ reinforces the idea that there is likely little (if any) change in energetic cost for bees flying in these conditions.

### Flight kinematics in headwinds versus tailwinds

The wind tunnel foraging experiments provided more detailed information about bees' flight paths and kinematics when flying in wind. Even at the moderate flow velocities used in our study, bees were far less likely to forage when wind was present (Fig. 2B); over 50% of the flights recorded in our first experiment (*n*=786 out of 1449 flights) occurred when there was no external flow, whereas less than 15% of flights (*n*=207) occurred with 2 m s^−1^ flow velocity, despite equal filming time across all flow conditions. Bees also displayed significantly higher path sinuosity with higher flow velocities, when flying both upwind and downwind (Fig. 2B), suggesting that flying in the presence of wind may cause bees to adjust their flight behavior. These results agree with a previous study showing that honeybees display higher lateral excursions when flying in the presence of wind (Burnett et al., 2022), and with the hypothesis that bees perform lateral oscillations to enhance the visual cues they use to control ground height (Baird et al., 2021), which may be particularly important when flying in wind. Alternatively, in the presence of wind, bees may simply be unable to maintain the straighter flight trajectories they adopt in still air.

Unlike the changes in path sinuosity, which occurred in both headwinds and tailwinds, we found that several measures of flight kinematics were significantly more variable only in tailwinds (Fig. 5). The standard deviation of body angle within individual flights (i.e. how much a bee pitched up and down during a flight) was significantly higher in 2 m s^−1^ tailwinds than in 0.75 m s^−1^ tailwinds or no flow, but there were no differences among flights in the upwind direction (Fig. 5A). The standard deviation of air speed within individual flights was significantly higher in 2 and 0.75 m s^−1^ tailwinds as compared with still air, and standard deviation of ground speed was higher in 2.0 m s^−1^ tailwinds than in 0.75 m s^−1^ tailwinds or no flow; for both of these variables, there were no significant differences among flights in the upwind direction (Fig. 5B,C). Because bees appear to control their air speed (and ground speed) by changing body angle (Fig. 4A), the increased variability in air and ground speed with tailwinds is likely due to increased variability in body angle under these conditions. Sample trajectories of flights in 0.75 and 2 m s^−1^ tailwinds illustrate this relationship; bees display rapid pitch-up maneuvers (Fig. 5D, top) that are associated with reductions in ground speed (Fig. 5D, bottom). The increased variability in body angle during flight in tailwinds may be due to increased body drag that bees experience at higher body angles and/or the active ‘braking’ maneuvers that bees perform to slow themselves down to their preferred ground speed (Movies 1 and 2).

Regardless of the cause, the increased variability in body angle and flight speeds that we found with mild tailwinds shows that flying downwind poses additional flight challenges that are not present when bees fly upwind, and this provides a possible explanation for our finding that bees prefer to fly upwind rather than downwind when given a choice (Fig. 1C). The increased variability in body angle and ground speed during flight in tailwinds may also result in less consistent optic flow information, which bees rely upon to control flight trajectory and determine the distance they have traveled.

### Implications for bees flying in natural environments

Our results suggest that flying downwind may impose a previously unrecognized challenge to bees foraging in natural environments, due at least in part to bees' strategy of maintaining a fixed ground speed during flight. If bees in open environments attempt to maintain constant ground speeds, and they rely on modulating body angle and generating negative (backwards) air speeds to maintain their ground speed, as in our study, the challenge posed by tailwinds would depend on the difference between the bee's preferred ground speed and the wind speed. Bees might be expected to encounter difficulties when flying downwind in winds that exceed their preferred ground speed by 1.0–2.0 m s^−1^ or more, as this would require bees to fly with negative (backwards velocities) of −1.0 m s^−1^ or more; for comparison, bees flying in 2 m s^−1^ tailwinds in our study had air speeds of −1.2 m s^−1^, and those flying in 2 m s^−1^ tailwinds in Baird et al.’s (2021) study had air speeds of approximately −1.7 m s^−1^. Given that wind speeds of 4.0–5.0 m s^−1^ are not uncommon in outdoor environments (classified as a ‘gentle breeze’ on the Beaufort wind scale; https://www.weather.gov/mfl/beaufort), flying downwind could pose a fairly regular flight challenge to bees in the wild.

Lower preferred ground speeds would likely cause greater difficulty in maintaining steady, downwind flight in the presence of tailwinds, but the preferred ground speed of bees in outdoor environments remains unclear. The ground speeds measured in our study align with previous findings that bees' preferred ground speeds are regulated by lateral optic flow and increase with tunnel width (i.e. with bees' distance from lateral obstacles), from less than 0.5 m s^−1^ in narrow tunnels up to approximately 2 m s^−1^ in 120 cm wide tunnels (Linander et al., 2016; Baird et al., 2021). Bees flying in cluttered outdoor environments, where they move through corridors of varying width formed by flowers, bushes, trees and other objects, might be expected to display fairly low preferred ground speeds, similar to those measured in lab wind tunnels. As a result, bees maneuvering through clutter may have difficulty flying downwind in even mild winds (e.g. 2–3 m s^−1^), whereas flying upwind at these flow speeds would pose no problem.

In corridors wider than 120 cm, or in the absence of lateral obstacles, bees switch to using ventral optic flow information from the ground to regulate their speed. In these cases, preferred ground speeds are likely to be higher than 2 m s^−1^, but the preferred ground speeds and actual air speeds of bees flying in natural, outdoor settings are largely unknown. Harmonic radar studies, in which long transponders attached to bees' thoraxes provide information about range (distance) and heading, report that honeybees display mean ground speeds of ∼3–3.6 m s^−1^ (Wolf et al., 2014; Capaldi et al., 2000) in outdoor environments. Some laboratory studies suggest that bees using ventral optic flow cues to regulate their speed prioritize maintaining constant optic flow, rather than maintaining constant ground speed. For example, honeybees adjust their height above the ground rather than their ground speed to maintain fixed optic flow when ventral flow cues are manipulated (Portelli et al., 2010).

In bumblebees, however, several studies suggest that ground speed and ground height may be controlled by two systems working in parallel, with different preferred optic flow set-points (Baird et al., 2021; Lecoeur et al., 2019). In a laboratory study, bumblebees maintained fixed ground speeds while flying in still air, headwinds of 1–2 m s^−1^, and tailwinds of 1–2 m s^−1^ (Baird et al., 2021), and adjusted their ground height depending on the flow direction, flying lower to the ground in headwinds (i.e. upwind) and higher in tailwinds (downwind). Because bees maintained the same ground speed in all conditions, these changes in ground height did not serve to maintain constant optic flow; instead, they likely increased variation in optic flow among conditions (Baird et al., 2021).

Field observations on honeybees and bumblebees also suggest that bees in the wild tend to fly closer to the ground when flying upwind and higher above the ground when flying downwind (Riley et al., 1999; Wenner, 1963). Because wind velocity approaches zero at the ground and increases exponentially with height (Stull, 1988), bees that fly lower to the ground in headwinds will drop down into an area with lower wind speeds. However, the reverse is true for bees flying higher above the ground in tailwinds: increasing ground height will cause them to encounter significantly faster wind speeds, which may increase the challenge of regulating either ground speed or ventral optic flow when flying downwind in natural environments. Although reliable estimates of outdoor ground speeds are lacking and the question of whether bees maintain fixed ground speeds when flying outdoors remains unresolved, mounting evidence suggests that bees avoid flying in wind whenever possible. Field studies on honeybees report that even when temperature and solar radiation levels are favorable, moderate wind speeds cause foraging activity to cease (Vicens and Bosch, 2000). Other studies report that the number of flower visits by bees drops sharply as wind velocity rises above 3 m s^−1^, ceasing entirely when wind reaches 4.5 m s^−1^ (Pinzauti, 1986). Similarly, a study on honeybees flying in a foraging arena with wind speeds of 0–3 m s^−1^ showed that honeybees visited fewer flowers with increasing wind speed, because of a significant increase in bees' hesitancy to take off when wind was present (Hennessy et al., 2000).

Thus, bees may sometimes choose to delay foraging trips until wind speeds decline; but in many cases, such as when resources in the hive are low or when wind picks up once bees are already away from the hive, bees will be forced to contend with flying in the presence of wind. We show here that bees are capable of maintaining constant, preferred ground speeds in the presence of mild tailwinds as well as headwinds, but they struggle to maintain consistent body angles and flight speeds when tailwind speed surpasses preferred ground speed (which requires bees to generate negative air speeds, flying backwards relative to the flow). Our results suggest that the challenge of maintaining controlled downwind flight with a fixed ground speed may be one reason why many bees are hesitant to fly in wind, and why they display a preference for flying upwind when given a choice. When bees do fly in tailwinds surpassing their preferred ground speed, the variability in body angle and ground speed that results may make the optic flow cues used for gauging flight distance less reliable. Alternatively, bees faced with a strong tailwind in the direction that they need to travel could choose a different route, flying crosswind, lower to the ground, or through clutter that may provide refuge from the wind. Bees could also stop attempting to regulate ground speed and allow themselves be pushed by the flow, but this would lead to the loss of optic flow cues used for distance calculations, which could have serious consequences (e.g. being unable to find their way back to the hive) in some situations.

Overall, our results suggest that rather than providing an energetic boost, tailwinds may impose a significant, underexplored flight challenge to bees foraging in the wild. In some cases, bees' inability to maintain consistent body angles and ground speeds when flying downwind could restrict their ability to fly in wind speeds well below their maximum, powered forward flight speed – a metric that has traditionally been used to define the flight boundary layer (Srygley and Dudley, 2008; Taylor, 1974), within which insects are assumed to be capable of controlled flight.

### Insights provided by technological advances

Our findings demonstrate the types of insights that can be gained from analyzing massive quantities of data collected from freely behaving animals – a task that has only become possible as computer power, video automation and deep learning techniques have become widely available over the past decade.

Journal of Experimental Biology (JEB) has played a key role in advancing our understanding of the biomechanics of animal locomotion, and of insect flight in particular, over the past century. Until recently, most research on insect flight biomechanics has focused on solving the puzzle of how insects fly. From the earliest proposed unsteady flight mechanisms (Weis-Fogh's ‘clap and fling’; Weis-Fogh, 1973), to studies exploring insect flight through flow visualization (e.g. Grodnitsky and Morozov, 1992; Bomphrey et al., 2005), analytical models (e.g. Dudley and Ellington, 1990; Wakeling and Ellington, 1997; Willmott and Ellington, 1997), computational fluid dynamics models (e.g. Liu et al., 1998; Sun and Tang, 2002; Miller and Peskin, 2004) and dynamically scaled robotic models (Sane and Dickinson, 2001, 2002; Birch et al., 2004; Birch and Dickinson, 2003; Maybury and Lehmann, 2004), JEB has published groundbreaking studies employing the newest techniques for understanding how insects generate and control aerodynamic forces.

Many of these studies were, by necessity, conducted in highly controlled laboratory environments, and were limited to analyzing or modeling one representative individual (and often a single wing stroke) for a given type of insect, because of both the time required for manual analysis and the limited computing power available. However, now that we have a basic understanding of how insects fly, and recent advances allow for the capture, storage and automated analysis of tens, hundreds or even thousands of flights in a single study, researchers studying insect flight biomechanics are free to explore a range of additional questions. Current research has expanded to questions exploring the wide variety of flight behaviors displayed by insects, and to understanding how and why flight biomechanics and behaviors vary – within individuals, between individuals and between species.

In order to fully explore these questions, particularly those concerning variability within and between individuals, it is necessary not only to collect large amounts of data but also to assign all data to uniquely identified individuals. Many past (and current) studies on insect flight avoid performing repeated measures by physically isolating each individual and collecting data during a single flight trial. This approach is valid for answering many types of questions, but sample sizes are limited by the time involved in manually testing individuals, and questions about within-individual variability (or about variable behaviors that require multiple trials to understand) cannot be answered with this single-trial approach. Repeated measures on known individuals over multiple days can be collected if individuals can be reliably distinguished from each other. This is typically accomplished by manually applying unique tags, which can be either visual (identified in camera/video images) or radio based (e.g. passive radio-frequency identification, or RFID, tags). Although tags are effective and useful for many studies, they may have some negative consequences on behavior (e.g. Switzer and Combes, 2016), and for many species, maintaining a fully tagged population with readable tags requires considerable effort (e.g. in bees, waxy build-up must be cleaned from tags, and the hive must regularly be anesthetized, and all individuals removed to tag newly emerged bees). In addition, many tags can only be identified over short distances – for example, passive RFID tags must pass within a few centimeters of a reader, and visual tags that can be automatically identified within images (i.e. those involving QR code-type identifiers) require high image resolution of the tags, and so are less useful for wide-field video data collected from larger flight arenas. The most promising new avenue for identifying individuals is using deep learning techniques to train computers to distinguish between individuals based on minor morphological differences (e.g. Murali et al., 2019), which eliminates the problems associated with applying, maintaining and reading tags. This method has not been widely tested and is not yet accessible to general users (i.e. to biologists rather than computer scientists), but it is under active development and is likely to become an important tool for biomechanics research in the coming decade.

Beyond the issue of uniquely identifying individuals, standard statistical tests performed on the large datasets that result from automated, high-throughput approaches to studying biomechanics must be interpreted with caution. Very large sample sizes are known to make relying on *P*-values as the sole measure of significance problematic, as *P*-values rapidly decline as sample size increases, leading to an increased risk of Type 1 statistical errors (i.e. ‘false positive’ results, in which the null hypothesis of no effect is erroneously rejected). To deal with this ‘*P*-value problem’ in large datasets, some researchers recommend reporting and relying more strongly upon effect sizes and confidence intervals than on *P*-values (Lin et al., 2013), and recent papers suggest alternative approaches, such as calculating a ‘decision index’ that explicitly considers the dependence of the *P*-value on sample size, and allows researchers to determine whether there is a ‘practical’ difference (i.e. a difference with actual, real-world implications) within a dataset (Gómez-de-Mariscal et al., 2021). Developing methods to analyze the statistical significance of large datasets is an area of ongoing research, which should be considered and incorporated (when possible) into future biomechanics research, as high-throughput techniques for collecting and analyzing data continue to be developed.

Despite the additional challenges to be addressed, adopting high-throughput approaches to data collection and analysis presents tremendous new opportunities for future research on insect flight biomechanics. In this study, by allowing bees to choose the flight conditions they prefer to traverse and automating our filming and analysis procedures to collect massive amounts of video data, we were able to identify significant patterns emerging from variable locomotory behaviors, and gain valuable insight into the biomechanics of flight in natural environments.

## Supplementary Material

10.1242/jexbio.245374_sup1 Supplementary information
