# Supplementary material for: Going against the flow: bumblebees prefer to fly upwind and display more variable kinematics when flying downwind
Source: J Exp Biol. 2023 Apr 18;226(Suppl 1):jeb245374. doi: 10.1242/jeb.245374 (PMC10263149; doi:10.1242/jeb.245374)
Supplement: Supplementary information [file jexbio-226-245374-s1.pdf]

Table S1. Results of two-sided binomial tests on daily flight preference tests (Experiment 1). Air flow in the two channels was in opposite directions; whether bees flew upwind or downwind depended on the tunnel they chose (left or right) and their flight direction (hive to feeder or feeder to hive). P-values less than 0.05 indicate that the proportion of flights in the upwind direction or in the right tunnel was significantly different from 0.5

Date	Flow speed in channels	Number of flights	Proportion upwind	p-value upwind	Proportion right	p-value right
6/15/17	1.25 m/s both	324	70.68	6.5×10^{-14}	58.02	0.0045
6/20/17	1.25 m/s both	92	63.04	0.016	55.43	0.35
6/7/17	1.25 m/s both	512	70.12	2.2×10^{-16}	52.54	0.27
6/11/17	1.25 m/s both	246	64.23	9.5×10^{-6}	48.78	0.75
6/9/17	1.07 m/s, 0.25 m/s	189	57.14	0.058	52.91	0.47
6/21/17	1.07 m/s, 0.25 m/s	164	59.15	0.023	41.46	0.035
6/14/17	1.07 m/s, 0.25 m/s	327	63.61	9.8×10^{-7}	59.63	5.9×10^{-4}
6/16/17	1.07 m/s, 0.25 m/s	315	61.27	7.5×10^{-5}	62.86	5.9×10^{-6}
6/8/17	1.07 m/s, 0.25 m/s	83	67.47	0.0019	50.60	1.00
6/12/17	1.07 m/s, 0.25 m/s	64	59.37	0.17	51.56	0.90
6/10/17	1.07 m/s, 0.25 m/s	332	66.57	1.6×10^{-9}	51.20	0.70
6/18/17	1.07 m/s, 0.25 m/s	281	69.75	3.0×10^{-11}	44.84	0.095

Journal of Experimental Biology • Supplementary information

Table S2. Flight variables measured in the wind tunnel experiments (Experiments 2 and 3). Data from Experiment 2 is shown on top and Experiment 3 on bottom. In these experiments, flights from the hive to the feeder were always in the downwind direction, and flights from the feeder to the hive were upwind. "SD" indicates the standard deviation of a variable, calculated within each flight trial and then averaged over all trials.

Flow velocity, flight direction	Number of flights	Path sinuosity	Mean ground speed (m/s)	SD ground speed (m/s)	Mean air speed (m/s)	SD air speed (m/s)
-2.0 m/s downwind	136	1.0217 ± 0.0243	0.890 ± 0.398	0.146 ± 0.081	-1.165 ± 0.383	0.147 ± 0.077
-0.75 m/s downwind	283	1.0148 ± 0.0194	0.912 ± 0.352	0.126 ± 0.068	0.152 ± 0.384	0.150 ± 0.087
0 m/s downwind	470	1.0087 ± 0.0119	0.901 ± 0.362	0.122 ± 0.068	0.901 ± 0.362	0.122 ± 0.068
0 m/s upwind	316	1.0185 ± 0.0190	0.757 ± 0.257	0.119 ± 0.065	0.757 ± 0.257	0.119 ± 0.065
0.75 m/s upwind	173	1.0279 ± 0.0253	0.767 ± 0.279	0.113 ± 0.070	1.503 ± 0.281	0.119 ± 0.082
2.0 m/s upwind	71	1.0320 ± 0.0275	0.693 ± 0.201	0.129 ± 0.066	2.670 ± 0.202	0.134 ± 0.071

Flow velocity, flight direction	Number of flights	Mean flapping frequency (Hz)	Mean body angle (deg)	SD body angle (deg)
-2.0 m/s downwind	32	188.2 ± 13.4	42.4 ± 9.5	5.9 ± 3.2
-0.75 m/s downwind	98	184.2 ± 14.0	39.8 ± 8.3	4.6 ± 2.7
0 m/s downwind	151	182.8 ± 16.2	33.8 ± 6.8	4.3 ± 2.1
0 m/s upwind	98	196.2 ± 12.1	27.7 ± 6.6	4.1 ± 2.2
0.75 m/s upwind	61	186.9 ± 15.1	22.0 ± 6.5	4.4 ± 2.8
2.0 m/s upwind	17	189.5 ± 13.2	20.8 ± 6.6	3.6 ± 1.6

Journal of Experimental Biology • Supplementary information

Table S3. Statistical testing for differences in flight variables with flow velocity. Variables were measured in the wind tunnel experiments (Expt. 2 and Expt. 3). In these experiments, flights from the hive to the feeder were always in the downwind direction, and flights from the feeder to the hive were upwind. The two different fight directions (upwind and downwind) were analyzed separately for differences between flow speeds. Results from a non-parametric Kruskal-Wallis chi-squared test are shown on top and for a one-way ANOVA, followed by post-hoc Tukey's HSD test, on bottom. "SD" indicates the standard deviation of a variable, calculated within each flight trial and then averaged over all trials.

Direction	Variable	Kruskal-Wallis chi-squared test	p-value
Downwind	Sinuosity	42.32	**6.5×10^{-10}**
Upwind	Sinuosity	24.08	**5.9×10^{-6}**
Downwind	Air speed	613.39	**$< 2.2 \times 10^{-16}$**
Upwind	Air speed	418.60	**$< 2.2 \times 10^{-16}$**
Downwind	Ground speed	1.17	0.56
Upwind	Ground speed	4.09	0.13
Downwind	SD air speed	9.43	**0.009**
Upwind	SD air speed	4.19	0.12
Downwind	SD ground speed	9.43	**0.009**
Upwind	SD ground speed	4.21	0.12
Downwind	Frequency	3.35	0.19
Upwind	Frequency	16.36	**0.0003**

Direction	Variable	One-way ANOVA	p-value	Tukey's HSD 0 vs. 0.75	Tukey's HSD 0 vs. 2.0	Tukey's HSD 0.75 vs. 2.0
Downwind	Sinuosity	F(2,886)= 34.9	**2.6×10^{-15}**	**5.2×10^{-6}**	**$< 1 \times 10^{-7}$**	**2.7×10^{-4}**
Upwind	Sinuosity	F(2,557)= 16.45	**1.1×10^{-7}**	**3.1×10^{-5}**	**1.3×10^{-5}**	0.38
Downwind	Air speed	F(2,886)= 1684	**$< 2 \times 10^{-16}$**	**0**	**0**	**0**
Upwind	Air speed	F(2,557)= 1724	**$< 2 \times 10^{-16}$**	**0**	**0**	**0**

Journal of Experimental Biology • Supplementary information

Downwind	Ground speed	F(2,887)= 0.177	0.84			
Upwind	Ground speed	F(2,557)= 1724	0.11			
Downwind	SD air speed	F(2,886)= 14.75	**4.9×10^{-7}**	**1.9×10^{-6}**	**0.0017**	0.91
Upwind	SD air speed	F(2,557)= 1.429	0.24			
Downwind	SD ground speed	F(2,886)= 6.122	**0.002**	0.69	**0.0015**	**0.021**
Upwind	SD ground speed	F(2,557) = 1.504	0.22			
Downwind	Frequency	F(2,278)= 1.677	0.19			
Upwind	Frequency	F(2,173)= 9.681	**0.0001**	**8.4×10^{-5}**	0.13	0.76

Journal of Experimental Biology • Supplementary information

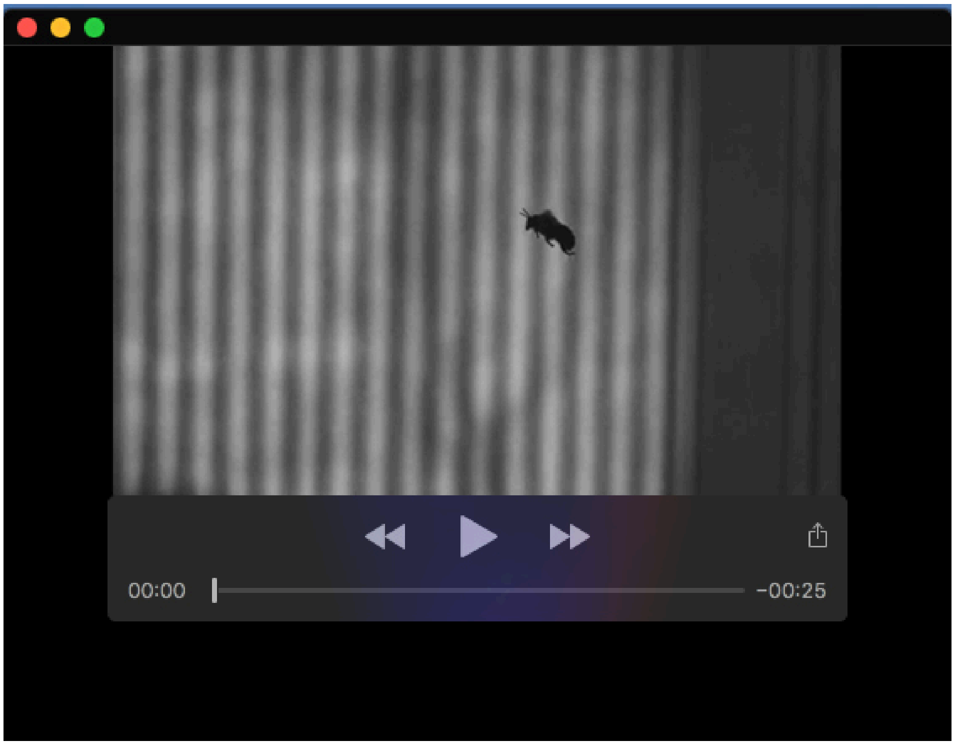

Movie 1. Lateral view of a bumblebee flying downwind with flow velocity of 0.75 m/s.

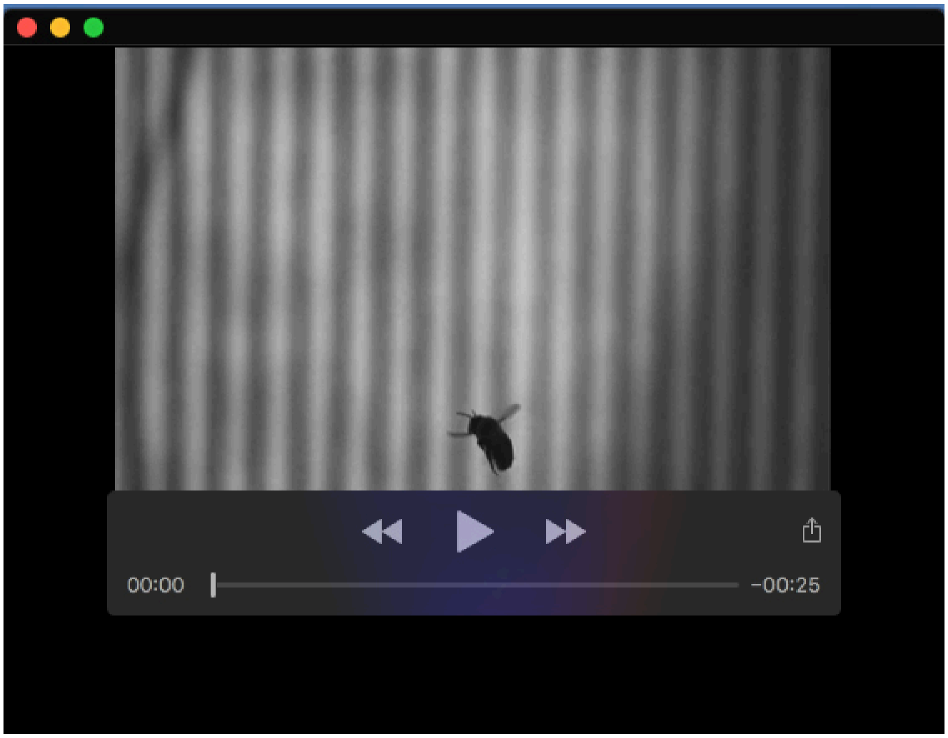

Movie 2. Lateral view of a bumblebee flying downwind with flow velocity of 2.0 m/s.

Journal of Experimental Biology • Supplementary information
